# Oblique Lateral Lumbar Interbody Fusion (OLLIF): Technical Notes and Early Results of a Single Surgeon Comparative Study

**DOI:** 10.7759/cureus.351

**Published:** 2015-10-15

**Authors:** Hamid Abbasi, Ali Abbasi

**Affiliations:** 1 Tristate Brain and Spine Institute; 2 Trinity College, University of Cambridge

**Keywords:** spinal fusion, lumbar spine, spine surgery, operative surgical procedures, minimally invasive surgery, interbody fusion, level 3 retrospective cohort study, disc disease

## Abstract

Background context: Lower back pain is one of the most prevalent and expensive health conditions in the Western world. The standard treatment, interbody fusion, is an invasive procedure that requires the stripping of muscles and soft tissue, leading to surgical morbidity. Current minimally invasive (MI) spinal fusions are technically demanding and suffer from technical limitations.

Purpose: Oblique lumbar lateral interbody fusion (OLLIF) is a new technique for fusion of the lumbar spine that overcomes these complications. Outcome measures include patient demographics, reported outcomes, and surgical outcomes.

Study design/Setting: Kambin's Triangle can easily be located as a silent window with an electrophysiological probe. Discectomy is performed through a single access portal with a 10 mm diameter. After a discectomy, the disc space is packed with beta-tricalcium phosphate soaked in autologous bone marrow, aspirated, and the cage is inserted. Finally, a minimally invasive posterior fixation is performed.

Methods: OLLIF’s major innovation is to approach the disc through Kambin’s Triangle, aided by bilateral fluoroscopy.

Results: We present data from 69 consecutive OLLIF surgeries on 128 levels with a control group of 55 consecutive open transformational lumbar interbody fusions (TLIFs) on 125 levels. For a single level OLLIF, the mean surgery time is 69 minutes (min) and blood loss is 29 ml. Surgery time was approximately twice as fast as open TLIF (mean: 135 min) and blood loss is reduced by over 80% compared to TLIF (mean: 355 ml).

Conclusions: OLLIF is a minimally invasive fusion that significantly reduces surgery times compared to open surgery. OLLIF overcomes the difficulties of traditional open fusions, making it a safe and technically less demanding surgery than open or minimally invasive TLIF.

## Introduction

Low back pain is one of the most prevalent and expensive health conditions in the Western world with up to 80% of all people suffering from it at some point during their life [1-2]. It is the leading cause of activity limitation for people under 45, one of the most common causes of health care utilization, and the third most common cause of surgical procedures in the United States [1]. In recent years, the rate of disability due to low back pain has increased dramatically, and consequently, costs have skyrocketed, particularly in patients with disc disorders [2-3]. In light of these data, improvements in surgical treatments of disc disorders could benefit hundreds of thousands of patients annually and contribute to lower health care costs.

As a treatment for disc disorders, posterior lumbar interbody fusion (PLIF) was developed in the 1950s and became the standard procedure to achieve interbody arthrodesis [4]. Harms, et al. developed the transformational lumbar interbody fusion (TLIF) technique [5]. TLIF has emerged as a popular alternative to PLIF because it allows for a unilateral approach, which leaves the contralateral laminae and facets available for posterior fusion. TLIF has been shown to cause less surgical morbidity and, thus, a quicker recovery than PLIF [6-7]. However, during the approach for TLIF, muscles are still detached and denervated, which may cause significant morbidity [8]. To address these issues, minimally invasive (MI) TLIF has been developed [9]. MI TLIF decreases blood loss and complication rates relative to open TLIF, but surgery times are similar or even longer than open TLIF [10-11]. Postoperative recovery is also reduced in MI TLIF and long-term outcomes are generally as good, but not better than for open TLIF [10, 12]. MI TLIF still requires a fairly generous laminectomy and facetectomy, so it is essentially a traditional open TLIF in which the spine is accessed and directly visualized through a smaller surgical corridor [9, 13]. This makes MI TLIF a technically challenging procedure that has not replaced open TLIF, even though it was developed almost a decade ago [13]. 

Oblique lateral lumbar interbody fusion (OLLIF) is a new technique that allows for the fusion of the lumbar spine through a single 10-15 mm incision, with faster surgery times and an easier approach than any previous technique. OLLIF eliminates the need for direct visual reference during surgery with the help of electrophysiological monitoring.

**Figure 1:**
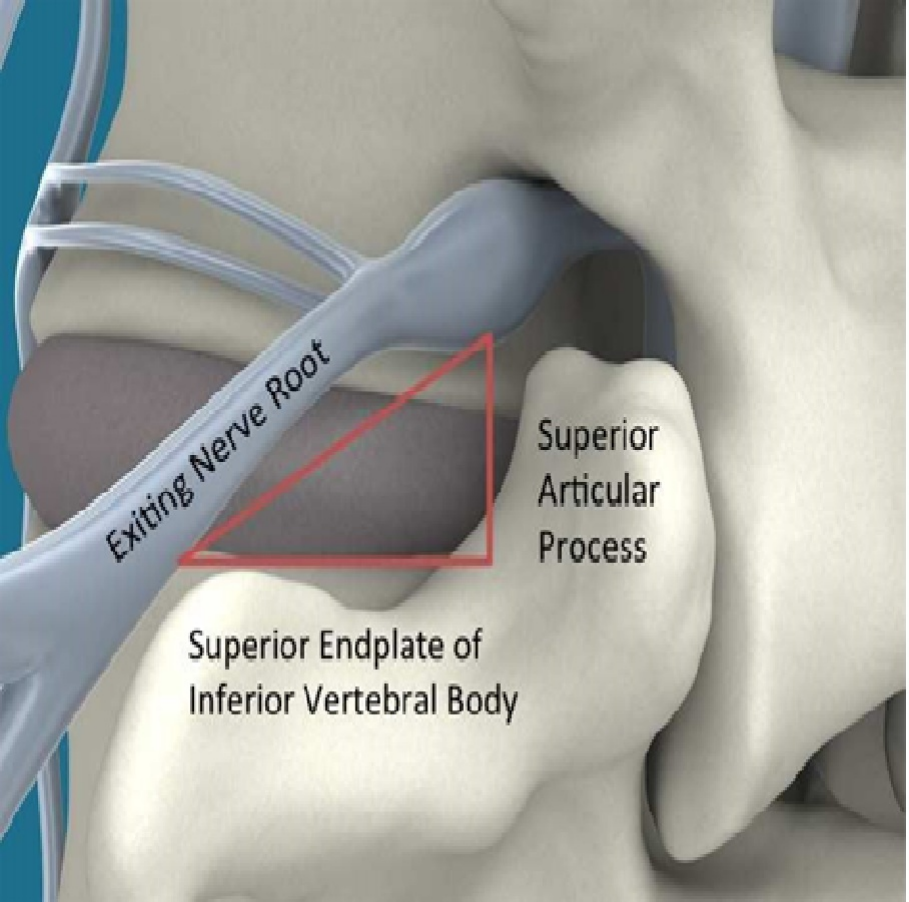
Kambin’s Triangle and surrounding anatomy

OLLIF’s major innovation is to perform spinal fusion via Kambin’s Triangle, as seen in Figure 1. Kambin’s Triangle has long been used as an access route to the disc [14-15]. It is made up of the exiting nerve (hypothenuse), the superior border of the caudal vertebra (base), and the traversing nerve root or the superior articular process (height) [16]. Because the triangle is an electrophysiologically silent window, OLLIF requires no direct visualization. The approach is guided solely through electrophysiological monitoring and biplanar fluoroscopy. In this article, we describe the technique and instrumentation used for OLLIF. We present perioperative outcome data from 69 OLLIF procedures and compare them to 55 open TLIFs done by the same surgeon.

## Materials and methods

### Study design

This was a retrospective case series including 69 OLLIF patients and 55 open TLIF controls. The exempt status of this study in accordance with FDA 21 CFR 56.104 and DHHS 45 CFR 46.101 regulations was approved by the Pearl Institutional Review Board (Indianapolis, IN 46260) in February 2015. The exception number assigned was 15-TRI-101.

### Surgical procedures

All procedures were done by the same surgeon as single surgeon procedures. The TLIF control group was selected from patients who underwent surgery before the surgeon started performing OLLIF to eliminate selection bias. All 124 procedures were performed in two Minnesota hospitals: the Douglas County Hospital, Alexandria, MN and Riverview Health, Crookston, MN. All surgeries were performed between March 2012 and December 2013. The study size derives from the number of surgeries in this time period.

Data were collected in the clinic electronic medical record (EMR) and summarized in Microsoft Excel. Mann-Whitney U-tests were utilized to test the null hypothesis that the OLLIF and TLIF groups have the same or identical mean distributions for age, BMI, blood loss, and the uncensored time duration variables. All data analyses were performed using SPSS (IBM SPSS Statistics for Windows, Version 22.0. IBM Corp., Armonk, NY).

### Outcome measures

Anesthesia/surgery times, blood loss, and fluoroscopy times were recorded for all patients by the clinic staff and entered into the EMR database immediately after surgery. Because no suction is used in OLLIF procedures, blood loss for the OLLIF group was measured by weighing sponges and subtracting dry weight. Routine follow-up was done within three months, six months, and nine to 12 months post-surgery.

### The OLLIF procedure

Patient Selection

Preoperative imaging included MRI, x-ray of the lumbar spine with flexion and extension, and in many cases, a discogram and computerized tomography (CT). OLLIF is indicated for severe degenerative disc disease, listhesis, discogenic stenosis, and disc re-herniation. With some experience, OLLIF can also correct for scoliosis and other deformities. All patients have gone through a full course of conservative therapy before being considered as candidates for surgery. Patient surgical indications are listed in Table 1.

**Table 1:** Patient surgical indications

Indication	n
Degenerative Disk Disease	61
Disk Herniation	14
Listhesis	22
Stenosis	15
Scoliosis	2

OLLIF is easiest in levels L1-L4 because rib attachments obstruct the approach in the thoracic spine, and for an L5-S1 fusion, the approach is sometimes obstructed by sacral ala and iliac crest. The following anatomical factors, initially considered for our indication setting, were relative contraindications for OLLIF: bony obstruction, significant spinal canal stenosis, large facet hypertrophy, Grade II listhesis, and other gross deformities. 

With some experience and modifications, OLLIF can still be performed for T12-L1 and L5-S1 fusions, or when some of the above contraindications are present. In our initial indication setting, an L5-S1 fusion was considered if the iliac crest was more than halfway towards the L5 vertebrae in the lateral x-ray view. Toward the end of the collection period, and with more surgical experience, L5-S1 fusions were more freely considered for OLLIF.

OR Setup

The patient is placed in the prone position on the operating table. To simplify the approach, the patient is tilted away from the surgeon by 3-5º until after the cage is inserted. Then, the patient is planed back into a true prone position. To enable quick readjustment, 3M Ioban transparent plastic draping (3M Center, St. Paul, MN) is used to help the surgeon get a good sense of the patient’s positioning.

Next, bilateral fluoroscopy is set up. The endplates of the target level should line up well in the lateral view. In the anterior-posterior (AP) view, the disc needs to be visible but not necessarily completely aligned, and the spinous process should be centered between the pedicles. Electrophysiological monitoring is set up on the major muscle groups and the skull. The somatosensory evoked potentials (SSEP) and electromyogram (EMG) are checked and monitored throughout the surgery.

Marking

**Figure 2:**
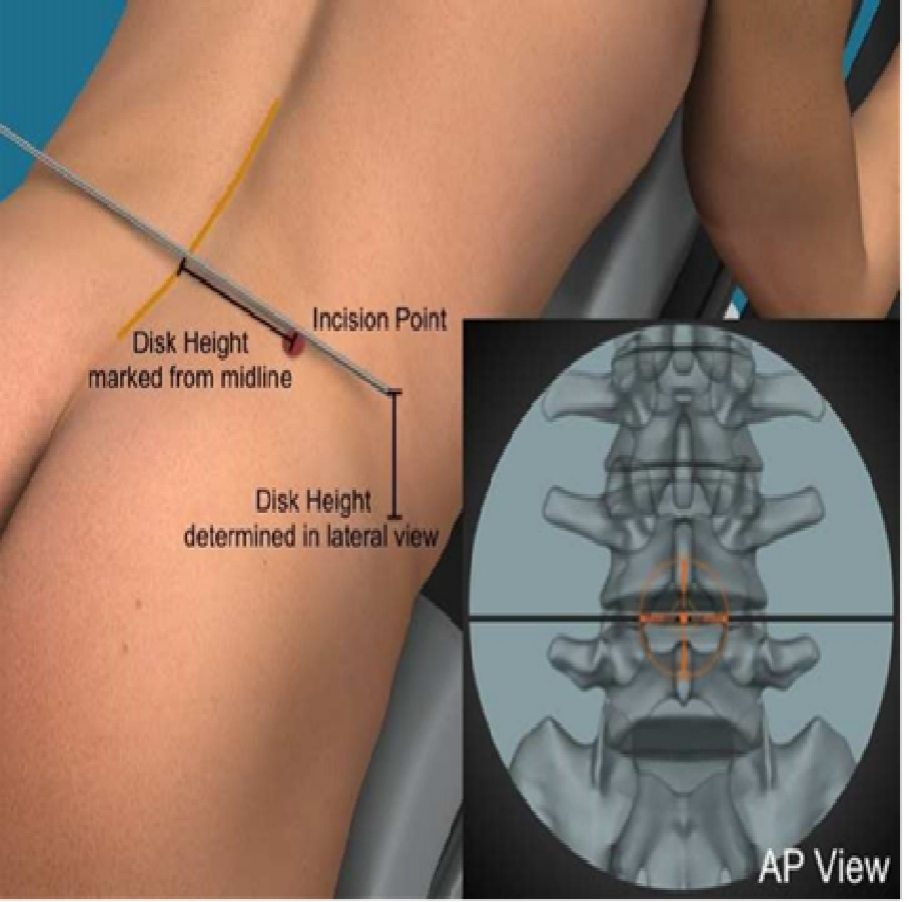
The incision point is chosen to give a 45º angle for approach to the spine

In the AP view, the midline and each disc is marked. A vertical line showing the midpoint of each disc is marked in the lateral view. To find the incision point, the depth of the disk in the lateral view is measured and marked as a distance from the midline in the AP view to give a natural 45º angle to the patient's back for approach, as illustrated in Figure 2. Usually, the incision is placed 10-13 cm from the midline. Multiple levels can be approached through the same incision by shifting the skin or approaching the disc at a slight angle.

Approach

**Figure 3:**
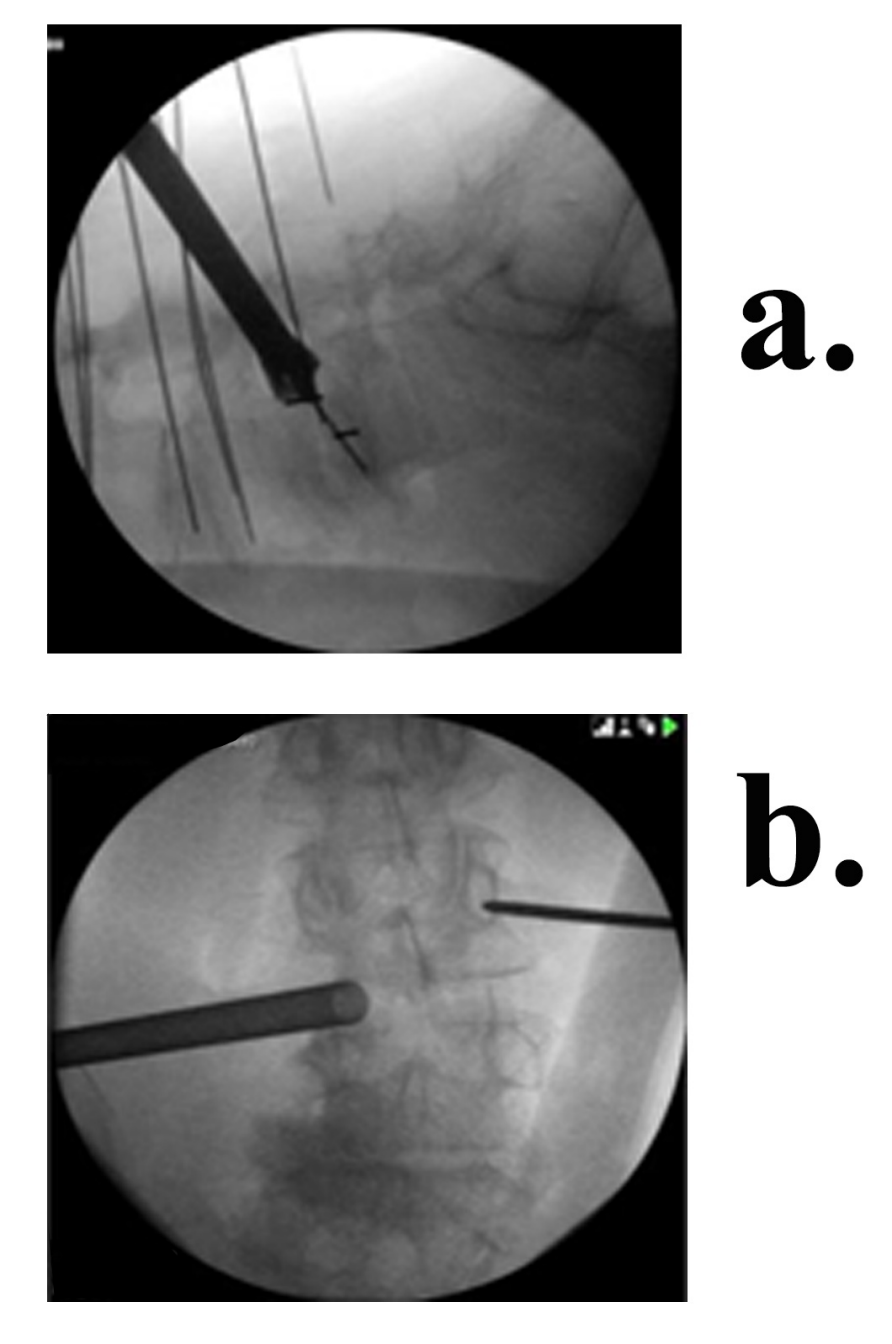
Fluoroscopic view of the access portal on the spine (a) Lateral (b) AP

**Figure 4:**
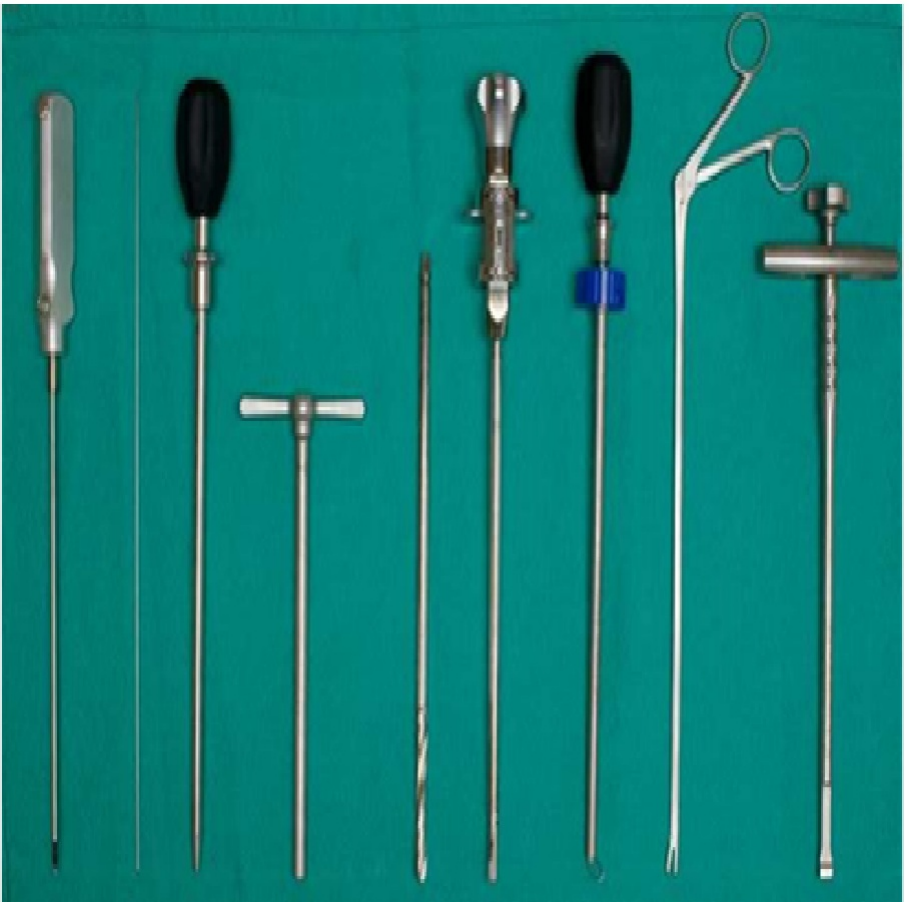
Instrumentation used for OLLIF, left to right: Probe, K-Wire, Dilator, Access Portal, Drill, Rotating Cutter, Disk Cutter, Pituitary, Cage Inserter

Aided by fluoroscopy, an electrode is inserted through the incision, traverses the retroperitoneal space, and pierces the iliopsoas fascia bluntly. To locate the silent window in Kambin’s Triangle, the electrode is stimulated at 3 mA. In some cases, especially when scoliosis or listhesis is present, the disc can be approached anterior to the nerve root, not actually passing through Kambin’s Triangle. This enables the surgeon to place the cage more strategically to achieve certain types of correction. Once contact with the disc is made, the electrode is stimulated at 4 mA to verify there is no contact with the nerve root [17]. Four of four twitches (no paralytics) is required for acceptance of the electrophysiological data. Once a silent window is found, the sleeve is pushed down over the electrode. The electrode is removed and a K – wire inserted past the midline into the disc. The sleeve is removed and a dilator introduced over the K-wire. While electrophysiological monitoring is continued, the dilator is entered with a careful circular motion, gently dilating the tissue. Often, a visible increase in foraminal size is observed at this point, which aids in the protection of the nerve-root. Once the dilator is entered into the disc space, the access portal is delivered over it and tapped into the disc until it is medial to the pedicle in the AP view and inside the disc space by about 1/2 cm in the lateral view. The final positioning of the access portal under bilateral fluoroscopy can be seen in Figure 3. The Zeus-O instrumentation system (Amendia, Inc., Marietta, GA), as displayed in Figure 4, was used.

Discectomy and Cage Placement

Disc material is removed first with a drill and then with a rotating cutter, ring curette, and long pituitary, all delivered through the access portal. The endplates are prepared with serial dilation of the rotating curette. Tactile feedback from the curette indicates when the endplates are reached. It is important to ensure the endplates are free. Light violation of the endplates is acceptable and slight bleeding is not uncommon at this point. Next, the disc space is packed with tricalcium phosphate (Berkeley Advanced Biomaterials Inc., Berkeley, CA) soaked in autologous bone marrow aspirate drawn from a Jamshidi needle in one of the pedicles.

A K-wire is placed and the access portal removed. At this stage, cage width and height can be determined using a trial spacer. However, with some experience, the cage dimensions can also be determined during discectomy through tactile feedback from the rotating cutter, which has markings that indicate how wide the blades are spread.

**Figure 5:**
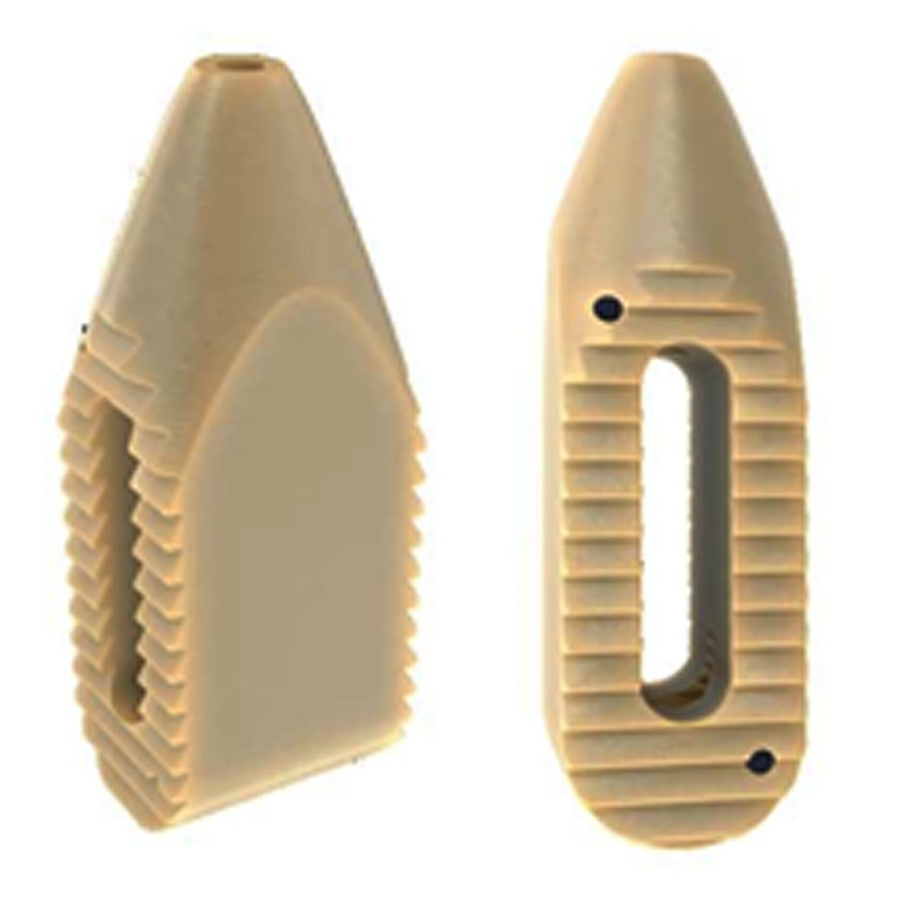
PEEK Zeus-O cage with bullet-nosed tip

**Figure 6:**
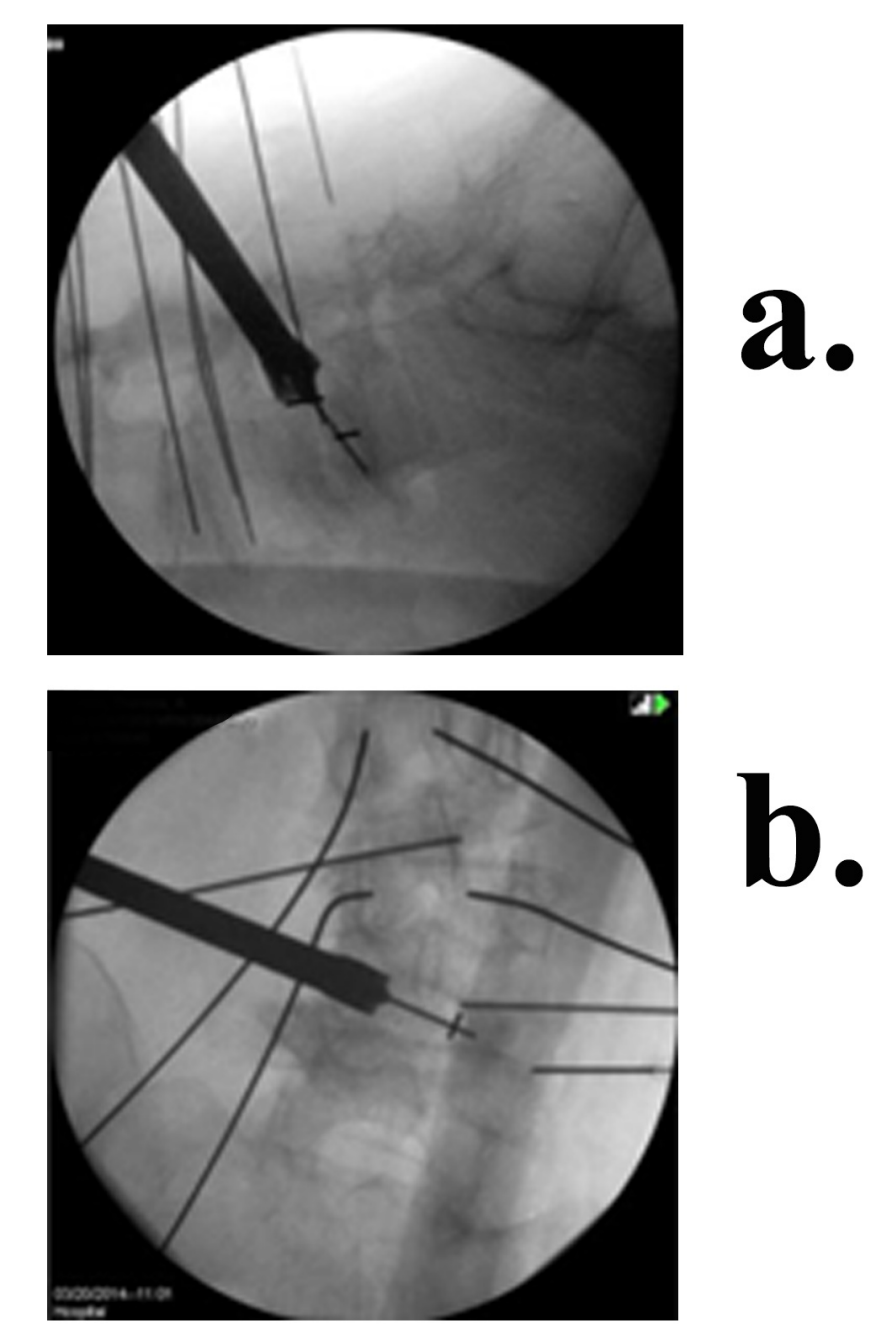
Fluoroscopic view of the cage being inserted (a) Lateral (b) AP

**Figure 7:**
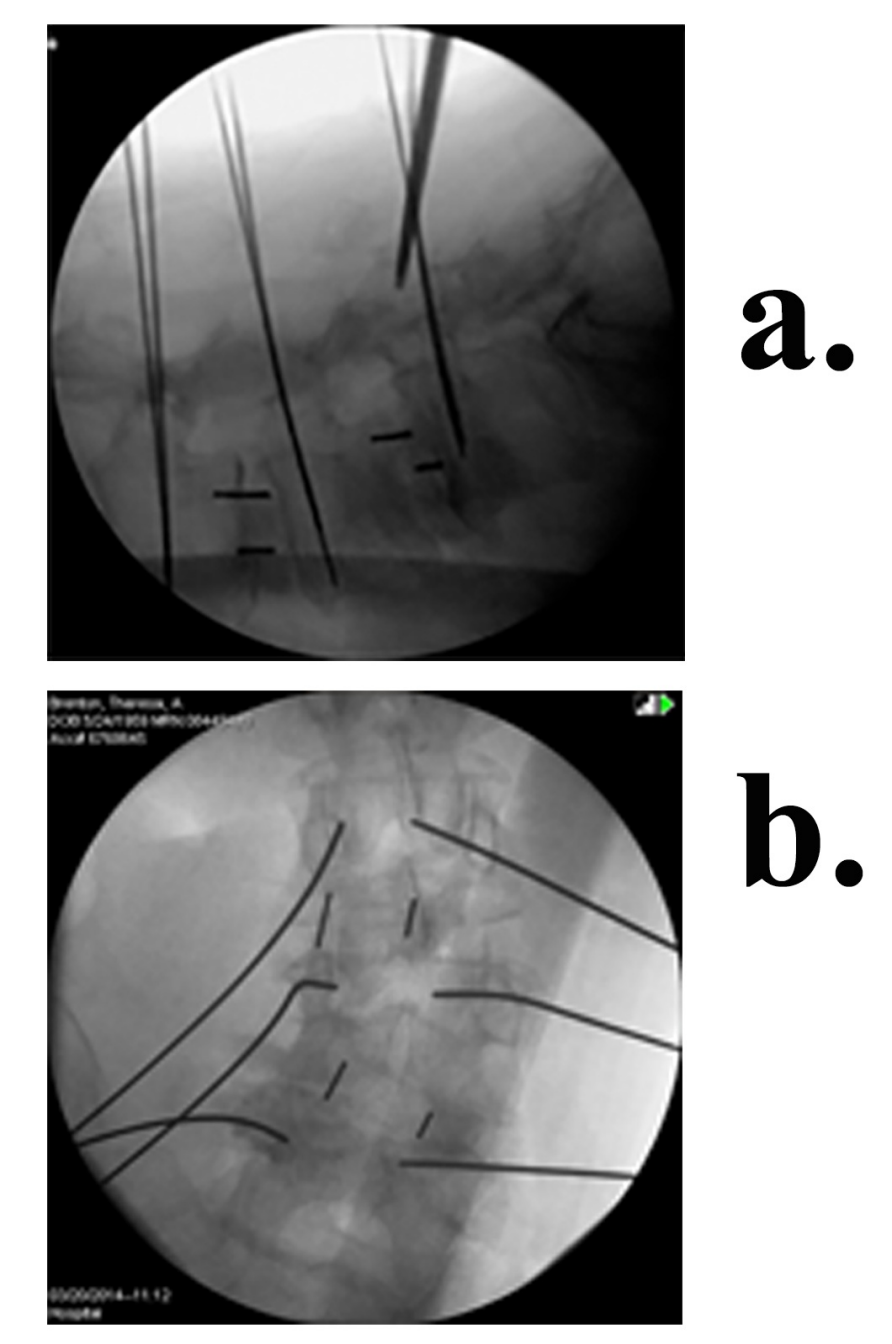
Completed 2 level OLLIF with cages marked by arrows, K-wires for fixation are also visible (a) Lateral (b) AP

Next, the cage (PEEK Zeus-O cage manufactured by Amendia) (Figure 5) is inserted over the K-wire aided by fluoroscopy as seen in Figure 6. Its conical shape assures that it makes its way through the muscle fascia and gently pushes the nerve root out of the way. For easier entry, the cage can be rotated by 90º during approach and de-rotated once the tip has entered the disc space. With mallet taps, the cage is entered until 1/3 of the cage is past the midline. Some electrophysiological activity is not unusual during cage entry. However, all activity subsides once the cage is placed because the cage increases pedicle distance and causes indirect foraminotomy. A fluoroscopic image of completed cage entry in a two level fusion is shown in Figure 7.

Posterior Pedicle Screw Fixation

After cage placement, all patients undergo percutaneous posterior pedicle screw fixation. Savannah-T posterior instruments and high top screws manufactured by Amendia were used. The technique is similar to what has been described by Foley in 2001, but the technique was modified to allow for posterolateral fusion, in addition to interbody fusion [18]. Jamshidi needles have already been placed in one level at the beginning of the surgery to allow tricalcium phosphate to be saturated with bone marrow aspirate. Once the cage is inserted, all pedicles are tapped with Jamshidi needles that are stimulated up to 30 mA to assure there is no contact to the neural structure. All stimulation results above 18 mA were accepted. It is necessary to ensure all Jamshidi needles are positioned correctly because repositioning is easiest at this point. K-wires are then placed through the Jamshidi needles, and once the positioning of all K-wires is confirmed, the AP fluoroscopic arm is removed to ease screw placement.

**Figure 8:**
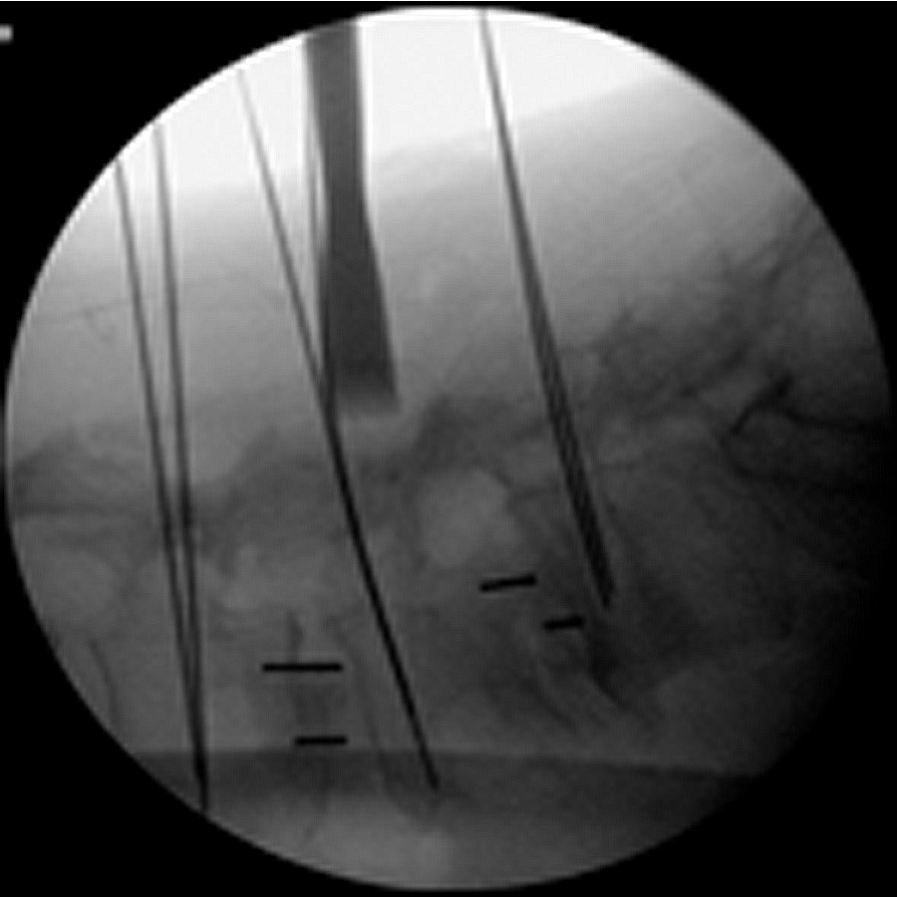
Lateral view of the osteotome during facet bareboning

**Figure 9:**
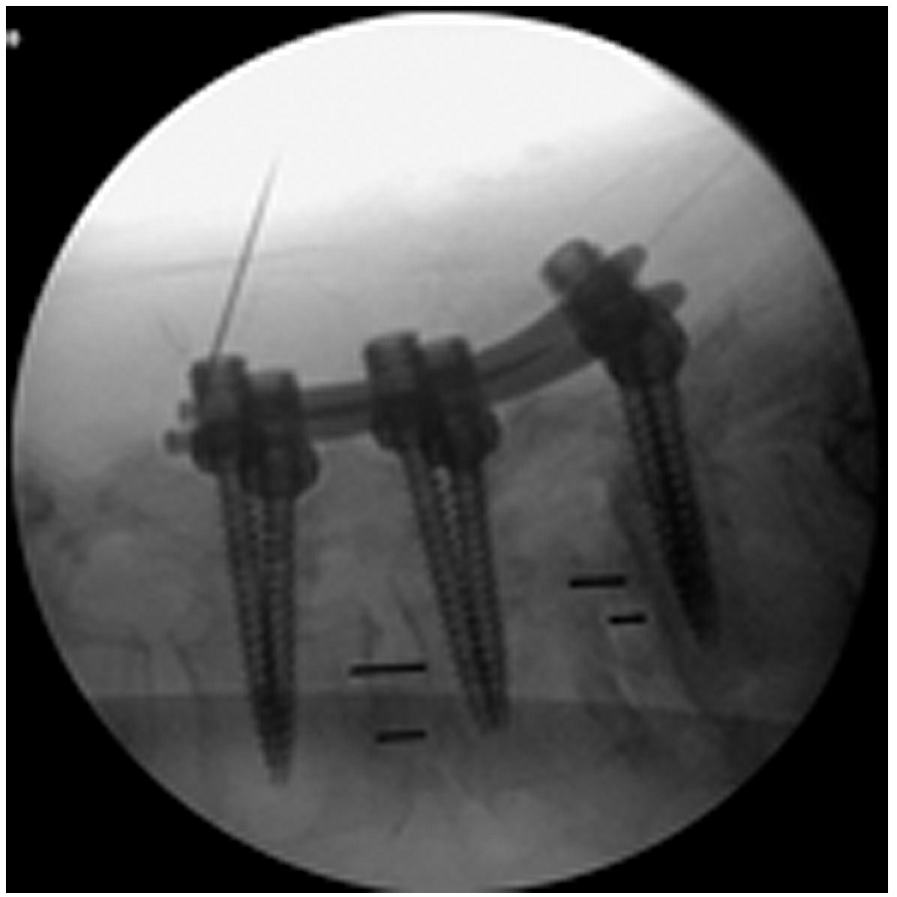
Lateral view of a completed 2-level OLLIF with posterior fixation

An osteotome with a groove is slid down the K-wire and the facets are bare boned aided by lateral fluoroscopy as illustrated in Figure 8. A small amount of dry tricalcium phosphate is placed in the just created space on the facets. To complete the surgery, the screws are inserted and the rod is placed, as described by Foley [18]. A fluoroscopic image of a completed two-level OLLIF with posterior fixation is shown in Figure 9.

## Results

Summary statistics for the two study groups are displayed in Table 2. There were no significant differences between the groups in either BMI or age. The only exception was that OLLIF 3 level patients were significantly older than their counterparts in the TLIF comparison group.

**Table 2:** Mean summary statistics of the study groups. ** Difference Significant at p<0.05

1 Level	OLLIF	TLIF	p value
Number	28	9	-
Age	56.1±15.2	64.1±20.9	0.226
BMI	29.5±5.8	31.0±4.8	0.541
2 Level	OLLIF	TLIF	p value
Number	24	19	-
Age	59.0±18.5	57.3±15.4	0.797
BMI	30.5±4.8	30.8±6.5	0.973
3 Level	OLLIF	TLIF	p value
Number	12	14	-
Age	68.7±11.7	58.3±8.7	0.023 **
BMI	30.9±8.4	35.4±6.3	0.16
4 Level	OLLIF	TLIF	p value
Number	4	9	-
Age	63.0±15.5	68.8±17.6	0.604
BMI	30.0±8.3	30.6±8.2	0.625

Perioperative outcomes are shown in Table 3. In all groups, OLLIF significantly reduced surgery times, blood loss, and hospital stay compared to TLIF. In the 1 level group, the mean hospital stay was reduced 1.6-fold, mean surgery time was reduced almost two-fold, and mean blood loss was reduced 12-fold. There was one exception in that there was no difference between the two groups in the length of hospital stay in the 3 level patient groups. Fluoroscopy times were significantly longer (4.6-fold) in the OLLIF group (Table 3).

**Table 3:** Mean perioperative outcomes of the study groups

1 Level	OLLIF	TLIF	p value
Blood Loss (ml)	29.4±17.2	355.0±131.5	<0.001
Surgery Time (min)	69.2±29.4	134.9±21.7	<0.001
Fluoro (seconds)	201.8±73.2	43.8±29.9	<0.001
Days to discharge	2.6±1.7	4.2±1.2	0.001
2 Level	OLLIF	TLIF	p value
Blood Loss (ml)	76.7±78.4	452.6±327.6	<0.001
Surgery Time (min)	107.1±42.0	175.3±39.3	<0.001
Fluoro (seconds)	247.4±79.4	65.1±57.3	<0.001
Days to discharge	3.3±1.1	5.8±6.5	0.01
3 Level	OLLIF	TLIF	p value
Blood Loss (ml)	69.3±57.6	618.6±353.9	<0.001
Surgery Time (min)	136.6±42.9	213.7±40.5	<0.001
Fluoro (seconds)	455.5±234.9	100.5±106.9	<0.001
Days to discharge	4.5±2.2	4.3±1.3	0.94
4 Level	OLLIF	TLIF	p value
Blood Loss (ml)	125.0±66.1	589.4±255.3	0.009
Surgery Time (min)	176.0±25.8	250.2±73.6	0.02
Fluoro (seconds)	524.3±243.3	88.2±75.1	0.006
Days to discharge	4.0±0.0	6.7±1.6	0.011

**Figure 10:**
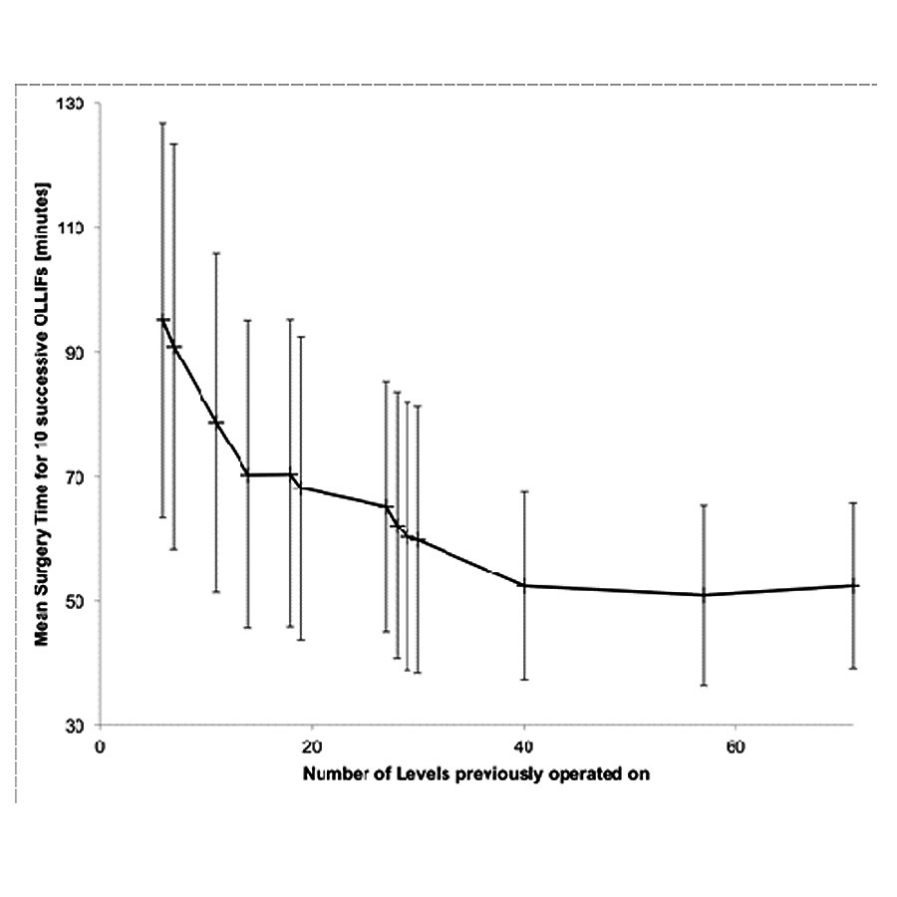
The surgeon’s learning curve. Values are displayed as mean ± SD of 10 successive single level OLLIF surgeries

Additionally, OLLIF was a straightforward procedure to learn. Figure 10 demonstrates how the mean surgery time for 10 successive OLLIFs dropped rapidly with experience. After operating on approximately 40 levels, the study surgeon came close to halving OR times for a single level OLLIF compared to his first surgeries.

## Discussion

A systematic review of studies on MI TLIF/PLIF by Goldstein, et al. found that single level surgery time for the MI fusions was not significantly different from their open equivalents [10]. Surgery times for single level MI TLIF/PLIF in Goldstein’s meta-analysis ranged from 104 to 390 minutes. This is much longer than our single level OLLIF cohort where surgery times ranged from 33 to 227 minutes. Goldstein et al. additionally reported blood loss ranging from 51 ml to 496 ml in the MI TLIF/PLIF subjects, which is also higher than the 5 ml to 300 ml reported in our single level OLLIF group [10].

OLLIF is the first spinal fusion that can be completed through a single 10 mm incision. Current MI TLIF methods require an incision of 20 mm or larger [9]. During approach for MI TLIF, muscles and soft tissue are stripped from the surgical corridor and a facet is removed, which exposes the patient to surgical morbidity [8-9]. OLLIF relies entirely on dilation and does not require facet removal. Whether this leads to lower rates of adjacent level disorder is currently under study.

In TLIF fusions, tricalcium phosphate is rarely packed into the disc space because of the risk of extrusion onto the neural structure. OLLIF allows the surgeon to freely pack the disc space with tricalcium phosphate or a biologic because the opening in the disc space is small and is essentially blocked by the cage. We hypothesize that this leads to higher fusion rates and are currently collecting data to investigate this.

Due to the conical shape of the Zeus-O cage (as illustrated in Figure 5), we were consistently able to place cages approximately 3-4 mm larger than during similar TLIF procedures. This creates a considerable opening of the foramen and even the spinal canal where disc herniation causes stenosis. Through the positioning of the cage and choosing the side of entry, it has even been possible to correct for listhesis and scoliosis.

This study is limited because it is a retrospective study. Using a retrospective patient cohort for comparison biases the data because clinical practices change over time. However, the data on perioperative measures, such as blood loss and OR time, was collected almost completely and clearly shows that OLLIF improves on TLIF. Due to the magnitude of this change, it is unlikely to be just a side effect of the study design or this particular surgeon’s skill. Therefore, OLLIF justifies further study as it has the potential to significantly improve the outcomes of patients with lumbar fusions.  

## Conclusions

This report describes OLLIF as the first MI fusion that is faster than open surgery. Data from 69 consecutive OLLIF surgeries and a control group of 55 consecutive open TLIFs suggest that OLLIF overcomes the difficulties of traditional open fusions, thereby making it a safe and technically less challenging surgery than open or minimally invasive TLIF.
